# Efficacy and prognosis of antiviral therapy on hepatitis C following treatment of lymphoma in HCV-positive diffuse large-cell lymphoma

**DOI:** 10.1007/s00277-017-3129-0

**Published:** 2017-09-22

**Authors:** Yutaka Tsutsumi, Chie Nakayama, Koki Kamada, Ryo Kikuchi, Daiki Kudo, Shinichi Ito, Satomi Matsuoka, Souichi Shiratori, Yoshiya Yamamoto, Hirohito Naruse, Takanori Teshima

**Affiliations:** 1 Department of Hematology, Hakodate Municipal Hospital 1-10-1, Minato-Cho, Hakodate, 041-8680 Japan; 2 Department of Gastroenterology, Hakodate Municipal Hospital, Hakodate, Japan; 3 Department of Hematology, Hokkaido Graduate School of Medicine, Sapporo, Japan

**Keywords:** HCV, Diffuse large B cell lymphoma (DLBCL), Direct-acting antiviral agents (DAAs)

## Abstract

The purpose of this study is to study the usefulness of post-remission antiviral therapy in cases of HCV-RNA-positive diffuse large-cell lymphoma. Antiviral therapy against HCV was performed after remission using CHOP or CHOP-like chemotherapy in combination with rituximab in five successive cases of HCV-RNA-positive diffuse large-cell lymphoma. The control groups consisted of a group of HCV-RNA-positive diffuse large-cell lymphoma cases prior to this trial (control 1), and a group of cases that tested negative for HIV, HCV, and HBV (control 2). All the cases were in remission at the time of initial treatment. There were no significant differences between the three groups in terms of age, sex, treatment, stage, or International Prognosis Index (IPI). When HCV antiviral therapy was performed after treatment for diffuse large-cell lymphoma, we observed no recurrence or deaths, and the 2-year overall survival and progression-free survival rates were significantly greater than those in the control 1 group (*P* = 0.0246). It is possible that a better prognosis can be achieved by performing HCV antiviral therapy after achieving remission in cases of HCV-RNA-positive diffuse large-cell lymphoma through the use of R-CHOP or similar treatments.

## Introduction

The hepatitis C virus (HCV) is considered to be one of the causative factors of liver cancer, and in HCV cases that have reached the stage of cirrhosis, the incidence of liver cancer is reckoned to be 23 to 35 times higher compared with that of healthy people [1, 2]. Therefore, controlling HCV to prevent the transition to cirrhosis is essential for suppressing the onset of liver cancer. On the other hand, the mechanism whereby HCV contributes to the onset of liver cancer is not yet clearly understood [2]. Although there have been reports suggesting the involvement of lymphoproliferative disease and HCV, there have been few pathological reports of a relationship between HCV and lymphoproliferative disease [3, 4]. Thus, although it is possible that HCV contributes to lymphoproliferative disease, there are still many uncertainties, including whether or not there is direct genetic involvement or if it contributes indirectly to the virus through chronic inflammation or the like, whether the high rate of recurrence is caused by the immune monitoring mechanism breaking down due to HCV infection, or whether therapeutic benefits are difficult to achieve [5]. We have confirmed that along with the rapid increase in HCV, there is a recurrence of B cell lymphoma and integration of HCV antibodies into lymphoma cells that were negative in the initial samples [6]. Also, when lymphoma specimens from HCV-positive lymphoma cases were stained with HCV-specific antibodies, 76.9% of the HCV-specific antibodies (including strong positive and weak positive) were positive in the lymphoma specimens. However, in this analysis, there was no difference in the strength of HCV-specific antibody staining in lymphoma specimens in cases of treatment resistance or recurrence [7]. This suggests an epidemiological possibility that HCV is associated with lymphoma. After giving anticancer treatment for HCV-positive and CD20-positive diffuse large B cell lymphoma, we administered HCV treatment according to the Japanese guidelines for the treatment of hepatitis C, and investigated whether there was an improvement in the prognosis of HCV-positive B cell malignant lymphoma.

## Patient and treatment

Focusing on cases where HCV antiviral therapy was performed following remission after chemotherapy, we conducted a prospective study of five cases of HCV-RNA-positive diffuse large-cell lymphoma that were registered during January 2015 through December 2016. Since this is a pilot study, we investigated whether or not the end points can withstand this treatment, and as secondary items, we set the 2-year progression-free survival (PFS) and overall survival (OS). All the registered cases were treated with CHOP therapy (rituximab 375 mg/m^2^ at day 1; vincristine 1.4/m^2^, doxorubicin 50 mg/m^2^, and cyclophosphamide 750 mg/m^2^ at day 2; prednisolone 60–100 mg/day at days 2–5) or R-THP-COP (rituximab 375 mg/m^2^ at day 1; vincristine 1/m^2^, pirarubicin 40 mg/m^2^, and cyclophosphamide 500 mg/m^2^ at day 2; prednisolone 40 mg/m^2^ at days 2–5). Although all cases achieved remission with the above treatment, in cases with a complete response or a complete response unconfirmed (CR, CRu), HCV genotype I cases were treated daily with daclatasvir 60 mg/day and asunaprevir 200 mg/day for 24 weeks, and HCV genotype II cases were treated with daily doses of ribavirin 200 mg/day and sofosbuvir 400 mg/day for 12 weeks.

## Quantitative detection and genotyping of HCV

HCV-RNA testing was performed using TaqMan HCV v 2.0 probes (Roche Molecular Systems, PL, USA) and genotyping analysis was performed by HCV Core Genotype (BEX Co LTD, Tokyo, Japan) using processes described by Ohno et al. [8]. A sustained virologic response (SVR) was defined as the absence of HCV-RNA 6 months after the end of treatment. An HCV viral load below the lower limit of detection was recorded as undetectable.

## Statistical analysis

**Fig. 1:**
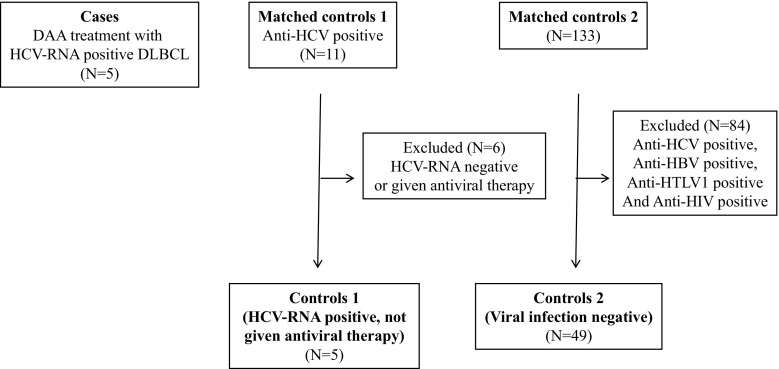
Flow diagram. DLBCL, diffuse large B cell lymphoma; HCV, hepatitis C virus; HBV, hepatitis B virus; HIV, human immunodeficiency virus

In this study, we calculated the 2-year disease-free survival rate and recurrence-free survival rate based on the time at which treatment for diffuse large-cell lymphoma was started. Out of 11 cases of HCV-positive diffuse large-cell lymphoma that were confirmed in this hospital’s medical records between January 2004 and 2015, comparative control groups were obtained by taking five cases that were HCV-RNA-positive and did not receive HCV antiviral therapy after achieving first complete remission by R-CHOP or R-THP-COP treatment (control 1), and 49 cases of diffuse large-cell lymphoma that were HBV/HCV/HIV-negative (out of 133 cases of diffuse large-cell lymphoma) where first complete remission was achieved with R-CHOP or R-THP-COP treatment (control 2) (Fig. 1). Subgroups were compared using *t* tests or Mann-Whitney tests for continuous variables and *χ* ^2^ or Fisher-exact tests for categorical variables. Pearson correlation tests were used to assess associations between continuous variables. The 2-year OS and 2-year PFS rates in these three groups were compared, with the differences evaluated using a stratified log-rank test. Finally, the Cox regression model was used to determine the association between HCV infection and OS or PFS. A *P* value below 0.05 was considered to be independently associated with the outcome. Statistical analyses were conducted with EZR. Our study was approved by the local ethics committee of Hakodate Municipal Hospital Institutional Review Board. Based on the Declaration of Helsinki, written informed consent was obtained from the patients.

## Results

**Fig. 2:**
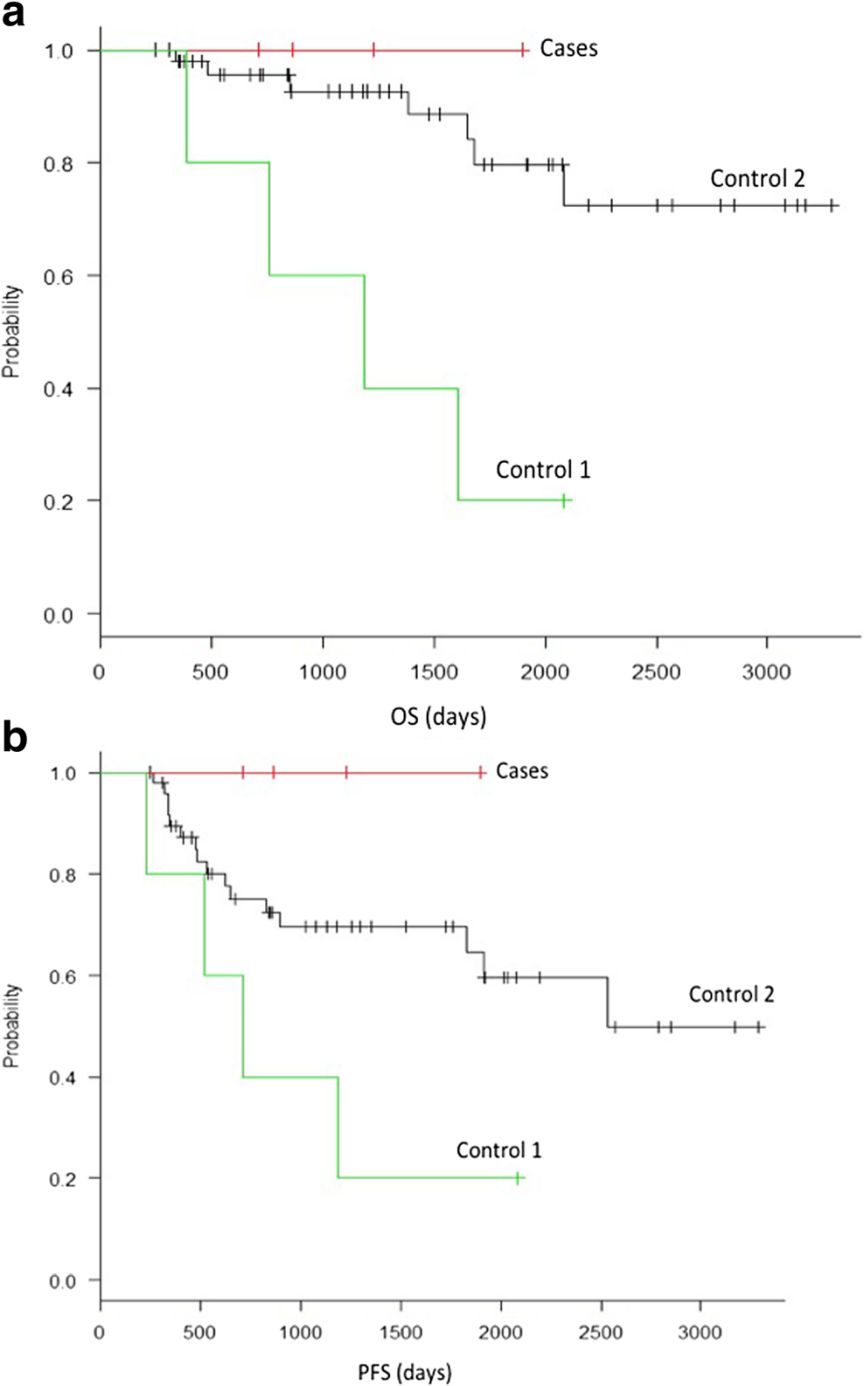
**a** Progression-free survival (PFS). All patients were free of recurrence following HCV antiviral treatment. PS was significantly better in patients given HCV antiviral treatment (*P* = 0.0246). **b** Overall survival (OS). All patients survived following HCV antiviral treatment. OS was significantly better in patients given HCV antiviral treatment (*P* = 0.063)

The patient details are shown in Table 1. The age of patients receiving direct-acting antiviral agent (DAA) therapy ranged from 63 to 75 years (median 65 years), while the control 1 group ranged from 55 to 80 years (median 73 years), and the control 2 group ranged from 39 to 85 years (median 64 years), there was no difference between the groups in terms of cancer staging, and there was no significant difference in the International Prognosis Index between the groups, even after dividing them into risk groups or low/low-int and int-high subgroups. Although treatment was performed using R-CHOP or R-THP-COP therapy in almost all cases, other treatments were used for three cases in the control group. There was no significant difference between the treatment groups in terms of age, sex, LDH, HCV-RNA, or sIL-2R. Radiotherapy tended to be added more often in the control 1 group, but this difference was not significant. Also, throughout the course of treatment, the patients in the DAA group and control 1 group either had cirrhosis as a complication, or did not undergo any transition to cirrhosis during the course of treatment. The control 1 group was analyzed using stored serum, and out of five cases, three were found to be of HCV-RNA genotype 1 while the others could not be analyzed, and there was a significant difference from the DAA control group (*P* = 0.0159). Out of five patients who received antiviral therapy, three had HCV genotype IIa and two had genotype IIb. All five of them were given ribavirin and sofosbuvir. There were no side effects such as nausea, diarrhea, abdominal pain or other gastrointestinal symptoms, anemia, leukopenia, thrombopenia or other hematotoxicity symptoms, general malaise, fever, weight loss, or rash, and there was no discontinuation of antiHCV drugs. A SVR was obtained in all cases where DAA therapy was administered, while patients who were not given antiviral therapy all remained positive for HCV-RNA. Figure 2 shows the progression-free survival (PFS) and overall survival (OS) rates. The 2-year PFS and OS rates showed no recurrence or deaths in cases where HCV antiviral therapy was used, while the control 1 group was significantly inferior to control 2 with regard to both PFS and OS (*P* = 0.04, OS 0.0016). Since this is an exploratory study, we directly compared the control 1 group with cases using antiviral therapy. Cases where antiviral therapy was used against HCV after 2-year PFS anticancer drug treatment were significantly better than the control 2 group (*P* = 0.0246), but although the OS rate also tended to be better, a significant difference was not observed (*P* = 0.063).

**Table 1:** Patients characteristics

		With DAA	Control 1	Control 2	*P* value
*n*		5	5	49	
Age		65 [63–75]	73 [55–80]	6.4 [30–85]	0.355
Sex (%)	F	3 [60.0]	3 [40.0]	243 [49.0]	1
	M	2 [40.0]	3 [60.0]	25 [51.0]	
IPI (%)	Low	2 [40.0]	1 [20.0]	13 [26.5]	0.866
	Low-int	1 [20.0]	1 [20.0]	11 [22.4]	
	High-int	0 [0.0]	2 [40.0]	8 [16.3]	
	High	2 [40.0]	1 [20.0]	17 [34.7]	
IPI (%)	Low, low-int	3 [60]	2 [40]	24 [49]	1
	High-int, high	2 [40]	3 [60]	25 [51]	
PS (%)	0	S [100.0]	3 [60.0]	36 [73.5]	0.737
	1	0 (0.0)	1 (20.0)	4 (8.2)	
	2	0 (0.0)	1 (20.0)	6 (12.2)	
	3	0 (0.0)	0 (0.0)	3 (6.1)	
Stage (%)	1	1 (20.0)	1 (20.0)	6 (12.2)	0.796
	2	1 (20.0)	1 (20.0)	8 (16.3)	
	3	1 (20.0)	0 (0.0)	5 (10.2)	
	4	2 (40.0)		30 (61.2)	
8 symptom (%)	A	4 (80.0)	4 (80.0)	33 (67.3)	1
	B	1 (20.0)	1 (20.0)	16 (32.7)	
sIL-2R		765 [416–2370]	814 [573–7470]	1470 [168–43,800]	0.635
LDH		220 [209–386]	186 [154–337]	271 [134–2159]	0.319
HCV-RNA (log IU/mL)		6.6 [5.9–7.0]	6.1 [4.8–6.5]		0.173
HCV genotype (%)	1	0 (0.0)	3 (60.0)		0.0159
	2a	3 (60.0)	0 (0)		
	2b	2 (40.0)	0 (0)		
	Unknown	0 (0.0)	2 (40.0)		
DAA (%)	Ribavirin + sofasbuvir	5 (100.0)			
Therapy (%)	Rituximab + CHOP or li regimen	5 (100)	5 (0)	46 (93.9)	
	Other regimen	0 (0)	0 (0)	3 (6.1)	
	with radiation	2 (40)	4 (80)	14 (28.6)	0.0627
	with autoPBSCT	1 (20)	0 (0)	4 (8.2)	0.619

*IPI* International Prognosis Index, *PS* performance status, *sIL-2R* soluble interleukin-2 receptor, *HCV* hepatitis C virus, *DAA* direct antiviral antigen

## Discussion

It was recently reported that performing antiviral therapy against HCV in the treatment of HCV-positive lymphoma has had positive effects including improvement of the lymphoma, and that antiviral therapy including interferon against low-grade lymphoma may be able to improve the prognosis when performed following chemotherapy for low-grade lymphoma [9, 10]. In our report, since HCV is often stained in tissue samples, and since cases where there is a drop in HCV viral RNA are less likely to recur [7], it is suggested that HCV is directly or indirectly associated with the occurrence and progression of lymphoma. However, the main causes of the transition to lymphoma are still unclear. Furthermore, it has been reported that improvements can be obtained with antiviral therapy alone (e.g., in cases of splenic marginal zone lymphoma), and it is expected that the use of antiviral therapy against HCV-positive lymphoma yields an improved prognosis [11–13].

On the other hand, studies of whether treatment of HCV with antiviral drugs after treatment for diffuse large-cell lymphoma contributes to the prognosis are being conducted either retrospectively or through a combination of retrospective and prospective methods [14, 15]. It has been reported that the 5-year OS and PFS can both be improved [14, 15]. Also, a report by Michot et al. pointed out the possibility of including cases where diffuse large B cell lymphoma is thought to have transformed from splenic marginal zone lymphoma [14], but in this case, cases that have transformed from splenic marginal zone lymphoma are not included. In Europe and the USA, there are many cases where splenic marginal zone lymphoma is associated with HCV. These cases are highly responsive to HCV antiviral therapy, suggesting the possibility of an improved prognosis. On the other hand, in a report by Michot et al., there were cases in which SVR could not be obtained in the antiviral therapy group, while in a report by Hosry et al., there were many cirrhosis cases among the analysis subjects, and it is thought that these factors may have acted on OS and PFS in a negative direction [14, 15]. In our analysis, genotype 2 was more common in the DAA group, while genotype 1 was more common in the control 1 group, and the difference in genotype may also have affected the outcome of treatment. It is thought that this affected the OS and PFS analysis of this report and earlier reports [14, 15].

Since DAA was used after obtaining confirmation of chronic HCV-positive hepatitis by liver biopsy in the cases analyzed here, DAA therapy was performed about 6 weeks after treatment with anticancer drugs. However, there are no clear guidelines on the appropriate timing for DAA treatment, such as whether antiviral therapy should be performed after or during lymphoma treatment, or before anticancer treatment of the lymphoma. A recent report investigated the interactions of DAA administered simultaneously with various anticancer drugs in cancer cases complicated by HCV infection. In this report, although blood toxicity and gastrointestinal toxicity were found, the change brought about by DAA therapy was only 10%, and the SVR was also 95%, and it was reported that it may be possible to use DAA therapy simultaneously with anticancer treatment [16, 17]. On the other hand, in this analysis, there were no problems arising from the continued use of DAA therapy, or complications requiring a change of medication regime, but this could be because there were no cases where DAA therapy was performed at the same time as the anticancer treatment.

In this study, we are conducting a prospective examination of five cases, and so far, 2 years has elapsed since the start of treatment, but in all five cases, the patients have survived without any recurrence, and it seems that following chemotherapy with antiviral therapy may contribute to the prognosis in cases of HCV-positive diffuse large-cell lymphoma. Furthermore, although analysis has only been performed in a few cases, it seems that HCV-RNA-positive cases may have a poorer prognosis than non-HCV-infected diffuse large-cell lymphoma cases, and although there are signs that the prognosis is improved for DAA treatment cases, it may be recommended that DAA treatment is performed after remission in HCV-RNA-positive diffuse large-cell lymphoma. However, since we have only examined a few cases for a short period of time, we are hopeful that the usefulness of antiHCV therapy will be demonstrated more clearly by a prospective study involving a larger number of people.
